# Comparison of L-Shaped and U-Shaped Beams in Bidirectional Piezoelectric Vibration Energy Harvesting

**DOI:** 10.3390/nano12213718

**Published:** 2022-10-23

**Authors:** Weile Jiang, Lu Wang, Xinquan Wang, Libo Zhao, Xudong Fang, Ryutaro Maeda

**Affiliations:** 1 School of Humanities and Social Science, Xi’an 710049, China; 2 State Key Laboratory for Manufacturing Systems Engineering, International Joint Laboratory for Micro/Nano Manufacturing and Measurement Technologies, Xi’an Jiaotong University (Yantai) Research Institute for Intelligent Sensing Technology and System, Xi’an Jiaotong University, Xi’an 710049, China; 3 School of Mechanical Engineering, Xi’an Jiaotong University, Xi’an 710049, China; 4 Shandong Laboratory of Yantai Advanced Materials and Green Manufacturing, Yantai 265503, China

**Keywords:** piezoelectric, energy harvesting, L-shaped beam, U-shaped beam, bidirectional

## Abstract

The traditional single degree of freedom linear piezoelectric vibration energy harvester (PVEH), such as the cantilever type, mainly works and resonates in a single direction and at a single frequency. To adapt broadband and bidirectional ambient vibration, this paper designs and compares two PVEHs of L-shaped beam and U-shaped beam through COMSOL simulation and prototype test. FEA modeling is introduced for accurate structure design with modal analysis, voltage frequency response analysis, and proof mass analysis with multiphysics electromechanical coupling simulation. Two PVEH prototypes with different gravity angles and clamping angles are tested at 0.1 g acceleration to find the optimal angle for maximum output power. The best clamping angle of L-PVEH is 135° with RMS power of 0.3 mW at 7.9 Hz, and that of U-PVEH is 45° with RMS power of 0.4 mW at 5.0 Hz. The proposed U-PVEH shows more advantages in low broadband and bidirectional vibration energy harvesting.

## 1. Introduction

The boom in wearable electronics and the Internet of Things has spawned the demand for distributed sustainable micro energy. There are various sources of kinetic energy and vibration energy in people and equipment. Vibration energy harvesting through piezoelectric materials and structures to generate power is a promising solution for self-power supply [1]. The traditional single degree of freedom linear piezoelectric vibration energy harvester (PVEH), such as the cantilever type, mainly works and resonates at the point of a single direction and single frequency. However, ambient vibration is often broadband and bidirectional, such as vehicle, train, airplane, and human motion [2]. For example, the vibration of trains and vehicles generally comes from the vertical and forward directions and the road irregularity excitation is also a random, broadband signal. Human walking and running also have vibration acceleration components in both forward and vertical directions. Therefore, the multi-directional and broadband PVEH is more suitable to address the actual demand to generate more power [3].

Scholars have proposed a variety of multidirectional energy harvesting structures [4]. Arranging the multi-branched piezoelectric beam structure along different directions is the basic configuration for multi-directional energy harvesting, such as double-branched beam [5] and dandelion-like multi-branched beam [6]. The drawback of multi-branched PVEHs is the low efficiency, because not all branches are working at the same time. Curved piezoelectric beams can overcome this problem in multidirectional energy harvesting, such as rainbow cantilever [7], bent cantilever [8], and arc-shaped cantilever [9]. However, the manufacture of curved piezoelectric beams is difficult.

Others use the multi-directional mass block structure of a single piezoelectric beam to realize multi-directional energy harvesting. Park J C [10] uses asymmetric inertial mass in a piezoelectric cantilever beam. Pendulum mass vibrates in multiple directions. Xu, J. [11] proposed an attached pendulum to the tip of a cantilever-type PVEH with internal resonance. In addition, the rolling mass can also realize multi-directional energy harvesting. Zhang, H. [12] used rolling steel balls to collide with piezoelectric sheets on four sides.

The folded beam structure is suitable for multi-directional and broadband energy harvesting, owing to the multimodal and high utilization of each piezo beam. The typical folded beam PVEHs include the L-shaped beam [13], V-shaped beam [14], U-shaped beam [15], and zigzag structure [16]. For broadening the frequency band of energy harvesting, Andò [17] uses a magnetically coupled bidirectional staggered piezoelectric cantilever.

The L-shaped beam PVEH has two degrees of freedom. Erturk et al. [18] proposed a linear distributed parameter model for a PVEH with an L-shaped structure, and studied the broadband piezoelectric output characteristics under 1:2 internal resonance of the system [19].

Ceponis, A., et al. optimized the L-shaped beam clamping angle. When the base has harmonic vibration in the horizontal and vertical directions, the maximum output power is 16.85 µW and 15.94 µW, respectively. Wei-Jiun Su et al. [20] present a U-shaped multi-modal bi-directional PVEH and investigate the influence of the aspect ratio of the U-shaped PVEH on the modal frequencies and voltage output.

To compare the L-shaped beam and U-shaped beam in bi-directional PVEH design, this paper investigates two PVEH designs by COMSOL simulation and prototype test. FEA modeling is introduced for accurate structure design with two modal analysis. Stress check and the stopper design method are proposed for the L-shaped PVEH and U-shaped PVEH, respectively. The influence of the proof mass on the resonant frequency and voltage response curves is also discussed in multiphysics electromechanical coupling simulation. A vibration experimental test is carried for the two PVEH prototypes. A comparison of the gravity angle and clamping angle is performed to find the optimal angle for maximum output power in bidirectional piezoelectric vibration energy harvesting.

## 2. Modeling and Simulation

### 2.1. FEA Modeling

**Figure 1:**
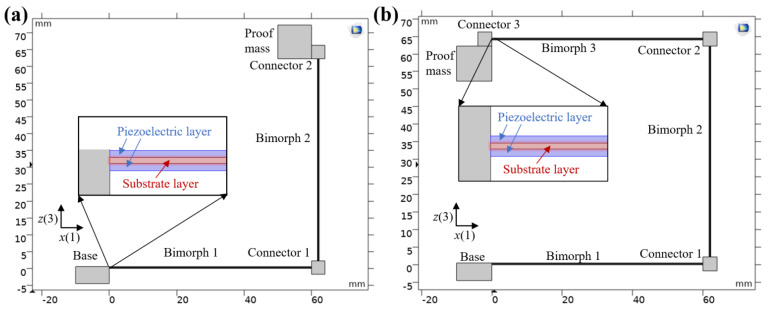
Schematic of PVEHs: (**a**) L-shaped PVEH and (**b**) U-shaped PVEH.

Two kinds of PVEHs are designed using the finite element method (FEM) by the commercial software COMSOL 5.5 (Burlington, MA, USA). In the process of geometric modeling, the three-dimensional modeling is simplified to two-dimensional modeling and the width direction feature is ignored, as shown in Figure 1.

The L-shaped PVEH is mainly composed of a fixed base, two piezoelectric bimorphs, a proof mass, and two connectors. Bimorph 1 is connected to the base and connected to bimorph 2 through connector 1, which maintains a right angle; bimorph 2 is connected to the mass block through connector 2.

The U-shaped PVEH is mainly composed of a fixed base, three piezoelectric bimorphs, a proof mass, and three connectors. Bimorph 1 is connected to the base and connected to bimorph 2 through connector 2, and the two maintain a right angle; bimorph 2 is connected to bimorph 3 through connector 2, and the two maintain a right angle; bimorph 3 is connected to the proof mass block through connector 3.

The piezoelectric bimorph is composed of a substrate layer and piezoelectric layers on both sides. The piezoelectric layer material is PZT-5H, the intermediate layer material is 4J36 alloy, and two piezoelectric layers of bimorph are connected in parallel with the same direction of polarization. The connector and base material are polymethyl methacrylate (PMMA), and the proof mass block material is copper. Its structural parameters are shown in Table 1.

### 2.2. Modal Analysis

**Figure 2:**
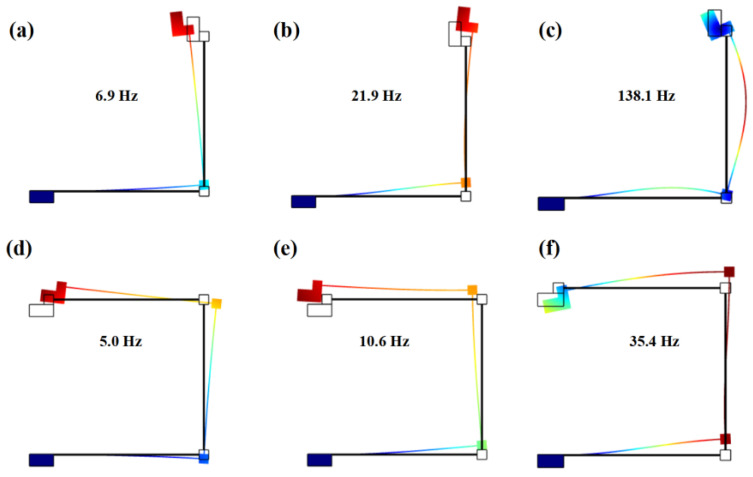
Modal analysis: (**a**) first modal, (**b**) second modal, and (**c**) third modal of the L-shaped PVEH; (**d**) first modal, (**e**) second modal, and (**f**) third modal of the U-shaped PVEH.

Model analysis in solid mechanics does not require electromechanical coupling. For the side length of the proof mass block of 10 mm and 5 mm, the L-shaped PVEH has three-order eigenfrequencies of 6.9 Hz, 21.9 Hz, and 138.1 Hz, respectively, as shown in Figure 2a–c. The U-shaped PVEH has three-order eigenfrequencies of 5.0 Hz, 10.6 Hz, and 36.7 Hz, respectively, as shown in Figure 2d–f. The U-shaped PVEH has a lower eigenfrequency and closer three-order modals compared with the L-shaped PVEH, because of the greater beam length.

### 2.3. Voltage Frequency Response Analysis

**Figure 3:**
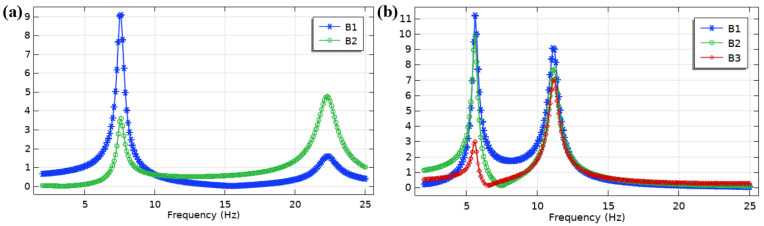
Open circuit voltage frequency response analysis results: (**a**) L-shaped PVEH and (**b**) U-shaped PVEH.

To explore the voltage frequency response of the two PVEHs in low frequency, electrostatic fields and circuits are added to the COMSOL model to form a completed piezoelectric multiphysics electromechanical coupling simulation. In the frequency domain analysis, the frequency is swept from 2 to 25 Hz at an acceleration of 0.1 g. Every bimorph is connected with a load resistance of 10 MΩ to obtain the open circuit voltage, as shown in Figure 3.

For the L-shaped PVEH, bimorph 1 (B1) has a higher peak voltage of 9.1 V than bimorph 2 (B2) in the first resonant frequency of 7.5 Hz. Bimorph 2 has a higher peak voltage of 4.9 V than bimorph 1 in the second resonant frequency of 22.3 Hz. For the U-shaped PVEH, bimorph 1 has the highest peak voltage of 11.2 V, while the voltage of bimorph 2 is close and that of bimorph 3 (B3) is the lowest in the first resonant frequency of 5.6 Hz. In the second resonant frequency of 11.2 Hz, the peak voltages of the three bimorphs are close, while bimorph 1 still has the highest peak voltage of 9.1 V.

Some conclusions can be drawn from the open circuit voltage frequency response curves: the PVEH resonant frequency is about 0.6 Hz higher than the eigenfrequency, because the electromechanical coupling leads to the increase in stiffness. Bimorph 1 has the highest peak voltage in the first two resonant frequencies for the L-shaped PVEH and U-shaped PVEHs, because bimorph 1 is closest to the fixed support end. Therefore, to avoid the complexity caused by the input of multiple piezoelectric bimorphs, only low-frequency vibration piezoelectric energy is harvested from bimorph 1 as far as possible. In the actual PVEH design, bimorph 2 and bimorph 3 are suggested to be replaced by metal beams to reduce costs.

The U-shaped PVEH has 2 V/Hz voltage of frequency in first resonant state, which is bigger than that of the L-shaped PVEH with 1.2 V/Hz. As the U-shaped PVEH resonance frequencies of the first two orders are close to each other, it is possible to design a nonlinear frequency extension to form broadband resonance. For example, the inclusion of stoppers alters the dynamics of the system and introduces nonlinearities with resonant frequency extension and a clipping effect. However, this FEA model has limitations in nonlinear dynamics interactive with stoppers. Methods for identifying the lumped parameters of the system and performing nonlinear analysis can be found in the literature [21].

### 2.4. Proof Mass Analysis

To explore the influence of the proof mass on the output, under the condition that the other parameters remain unchanged, the size of the mass is controlled by changing the side length of the square mass block, setting the side length to 5 mm, 7.5 mm, 10 mm, 12.5 mm, and 15 mm, respectively. Using the method in Section 2.3, the PVEH voltage frequency response curves with different proof mass are simulated to find the first-order and second-order resonant frequency, as well as their corresponding maximum voltage in open circuit state (*R_L_* = 10 MΩ).

**Figure 4:**
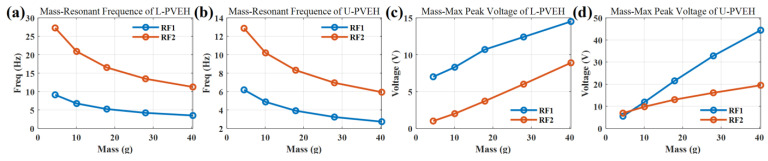
Simulation curves of mass-resonant frequency of the (**a**) L-shaped PVEH and (**b**) U-shaped PVEH at an acceleration of 0.1 g. Simulation curves of mass-max peak voltage of the (**c**) L-shaped PVEH and (**d**) U-shaped PVEH at an acceleration of 0.1 g.

The simulation results are shown in Figure 4. When the mass increases, the first-order and second-order resonant frequencies decrease, and the maximum output increases gradually. The comparison between the L-shaped PVEH and U-shaped PVEH shows that, when the mass block is large, the maximum output voltage and output power of the U-PVEH are much higher than those of the L-PVEH, and the first-order and second-order resonant frequencies of the U-PVEH are lower than those of the L-PVEH. When the mass block is small, although the resonant frequency of the U-PVEH is still less than that of the L-PVEH, the maximum output of the two is not very different. However, when the proof mass block is too small, the second-order output voltage of the U-shaped PVEH is bigger than the first-order output voltage.

## 3. Experiment and Discussion

### 3.1. Experimental Configuration

**Figure 5:**
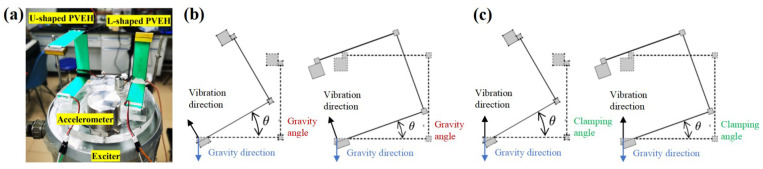
(**a**) Prototype of PVEHs. (**b**) Schematic of the gravity angle. (**c**) Schematic of the clamping angle.

Prototypes of the PVEHs are shown in Figure 5a; PVEH geometric design parameters are shown in Table 1. The PMMA for the base, connector, and stopper is fabricated by laser cutting. Similarly, for simulations in COMSOL with the side length of the proof mass block of 10 mm and 5 mm, a copper block with a weight of 8.96 g is bonded as the tip proof mass in the experiment. All of the parts are assembled by glue (ergo 5800). The vibration experimental platform and test method and the material of the PZT-5H piezoelectric bimorph are the same as in [22].

Based on the low and broadband vibration excitation from humans and vehicles, the experimental frequency sweep range is set to 2–15 Hz, the acceleration is set to 0.05 g, and the sine signal is 0.1 g. The vibration in the forward and vertical direction is the main concern of the experiment.

To compare the bidirectional energy harvesting characteristics of the L-shaped PVEH and U-shaped PVEH, the influence of gravity angle and clamping angle on output voltage and power is explored in the vibration experiment. The gravity angle is defined as the angle between the vibration direction and gravity direction, as shown in Figure 5b. The clamping angle is defined as the angle between the vibration direction and first bimorph length direction, as shown in Figure 5c.

### 3.2. Gravity Angle Comparison

**Figure 6:**
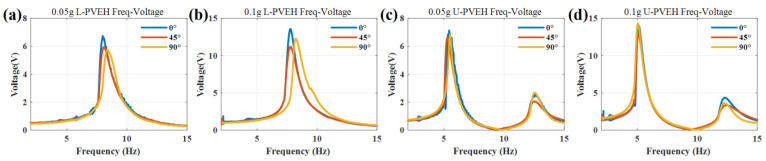
Experimental voltage frequency response curves with different gravity angles: the L-shaped PVEH at an acceleration of (**a**) 0.05 g and (**b**) 0.1 g; the U-shaped PVEH at an acceleration of (**c**) 0.05 g and (**d**) 0.1 g.

The gravity angle can be adjusted by changing the angle between the exciter axis and gravity direction. The clamping angle is fixed at 90° and the angle of the exciter is adjusted to be 0°, 45°, and 90°, respectively. The curve of output voltage with frequency under 0.05 g and 0.1 g excitation is measured, respectively, and the results are shown in Figure 6.

For the U-shaped beam, the change in gravity direction has no effect on the eigenfrequency and almost no effect on the output voltage. There is only a gap between the outputs near the second-order eigenfrequency point, with a maximum difference of 1 V.

For the L-shaped beam, the change in gravity direction has little effect on the eigenfrequency and output voltage. The included angle increases from 0° to 90°. The resonant frequencies of and under 0.05 g and 0.1 g excitation acceleration increase by 5.14% and 6.02%, respectively. The output voltage is reduced by 12.4% and 17.6%, respectively. These effects come from the change in the balance position of the vibration system caused by gravity and the experimental error.

### 3.3. Clamping Angle Comparison

The excitation direction can be changed by changing the clamping elevation between the piezoelectric beam and the plane of the exciter. The supporters are made by 3D printing, and the clamping angle of the piezoelectric beam is changed to 0°, 45°, 90°, and 135°, respectively. The output voltage curve with frequency under the excitation of 0.05 g and 0.1 g, as well as the RMS power curve with load resistance under the excitation of 0.1 g, are measured in a vibrating platform.

**Figure 7:**
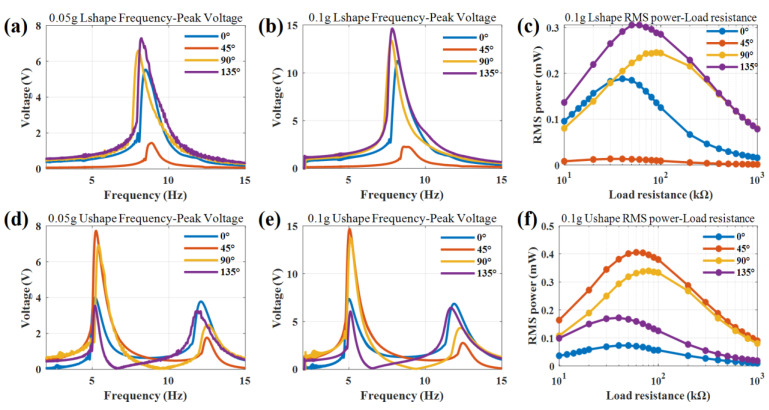
Experimental voltage frequency response curves with different clamping angles: the L-shaped PVEH at an acceleration of (**a**) 0.05 g and (**b**) 0.1 g; the U-shaped PVEH at an acceleration of (**d**) 0.05 g and (**e**) 0.1 g. Experimental curves of RMS power with load resistance with different clamping angle at an acceleration of 0.1 g: (**c**) the L-shaped PVEH at 7.9 Hz; (**f**) the U-shaped PVEH at 5.0 Hz.

As shown in Figure 7, the excitation direction has a great influence on the output voltage and RMS power and little influence on the resonant frequency within 5%. The output voltages are just proportional to the acceleration compared with 0.05 g and 0.1 g acceleration. For the L-shaped PVEH at 0.1 g acceleration, the maximum output voltage of 14.6 V appears at 135° in the resonant frequency of 7.9 Hz, which increases by about 30% compared with that of 11.23 V at 0°. When the minimum output voltage of 2.24 V appears at 45°, the relative decrease is about 73% compared with that at 0°. The maximum output RMS power of 0.3 mW occurs at 135° with the optimal load resistance of 60 kΩ.

For the U-shaped PVEH at 0.1 g acceleration, there are two resonant frequencies. For the first resonant frequency of 5.0 Hz, the maximum output voltage of 14.6 V appears at 45° and the maximum output RMS power is 0.4 mW with the optimal load resistance of 60 kΩ. For a clamping angle of 0° and 135°, the peak voltages in the first resonant frequency and second resonant frequency are almost same, at around 7 V, so the dual-modal exhibits broadband characteristics.

According to the bending moment theory, the projection distance between the center of gravity of the structure and the fixed support excitation point perpendicular to the vibration direction is the key factor affecting the output voltage. The farther the distance, the greater the moment of inertia, and the greater the piezoelectric voltage and power. The gravity angle does not change this projection distance, but the clamping angle does. In the actual design, the ratio of forward and vertical vibration accelerations should be considered to optimize the clamping angle to obtain the maximum output.

## 4. Conclusions

Two PVEHs with an L-shaped beam and U-shaped beam are designed and compared in this paper through COMSOL simulation and prototype test. Modal analysis showed that the U-shaped PVEH has a lower eigenfrequency and closer two-order modals compared with the L-shaped PVEH. Voltage frequency response analysis indicated that bimorph 1 has the highest peak voltage in the resonate state. Proof mass analysis showed that the resonant frequency decreases and the mass-max peak voltage increases with the increase in mass. The increase rate of the first resonant frequency with the mass of the U-shaped PVEH is larger than that of the L-shaped PVEH. The maximum voltage of the first two resonant frequencies can be adjusted by proof mass to the same value for the U-shaped PVEH.

Prototypes of the PVEHs are tested in sine vibration experiment with different gravity angles and clamping angles. The experimental results show that the direction of gravity has almost no influence on the output of PVEHs, while the clamping angle is the main factor affecting the output of PVEHs. The best clamping angle of L-PVEH is 135° with RMS power of 0.3 mW at 0.1 g acceleration at a resonant frequency of 7.9 Hz. The best clamping angle of U-PVEH is 45° with RMS power of 0.4 mW at 0.1 g acceleration at a resonant frequency of 5.0 Hz. The U-PVEH has a higher peak output, with 2.92 V/Hz and 0.08 mW/Hz, than the L-PVEH, with 1.85 V/Hz and 0.038 mW/Hz, in the experiment. Altogether, the proposed U-PVEH shows more advantages in low broadband and bidirectional vibration energy harvesting.

## Figures and Tables

**Table 1:** Design and simulation parameters of PVEHs.

Symbol	Parameters	Value
*l_b_*	Bimorph length	60 mm
*b*	Bimorph breadth	20 mm
*t_p_*	Piezoelectric layer thickness	0.18 mm
*t_s_*	Substrate layer thickness	0.14 mm
*l_m_*	Mass length	10 mm
*t_m_*	Mass thickness	5 mm
*l_c_*	Connector length	4 mm
*m_t_*	Tip proof mass	8.56 g
*Y_p_*	Piezoelectric layer elasticity	41.2 GPa
*Y_s_*	Substrate layer elasticity	110 GPa
*e*_31_	Piezo stress coefficient	−15.65 C/m^2^
*C_p_*	Piezoelectric capacitance	340 nF

## Data Availability

The data that support the findings of this study are available from the corresponding author upon reasonable request.
